# Trunk muscle dysfunction in patients with myotonic dystrophy type 2 and its contribution to chronic low back pain

**DOI:** 10.3389/fneur.2023.1258342

**Published:** 2023-10-26

**Authors:** Daniela Vlazna, Peter Krkoska, Michaela Sladeckova, Olesja Parmova, Tamara Barusova, Karolina Hrabcova, Stanislav Vohanka, Katerina Matulova, Blanka Adamova

**Affiliations:** ^1^Department of Neurology, Center for Neuromuscular Diseases (Associated National Center in the European Reference Network ERN EURO-NMD), University Hospital Brno, Brno, Czechia; ^2^Faculty of Medicine, Masaryk University, Brno, Czechia; ^3^Department of Rehabilitation, University Hospital Brno, Brno, Czechia; ^4^Department of Public Health, Faculty of Medicine, Masaryk University, Brno, Czechia; ^5^Institute of Biostatistics and Analyses Ltd., Brno, Czechia

**Keywords:** myotonic dystrophy type 2 (MD2), function tests, neuromuscular diseases (NMD), low back pain, paraspinal muscles, muscle strength, muscular endurance, respiratory muscles

## Abstract

**Introduction:**

Myotonic dystrophy type 2 (MD2) presents with a varied manifestation. Even though the myopathy in these patients is more widespread, axial musculature involvement is one of the most prominent conditions. MD2 patients also often report chronic low back pain (CLBP). The purpose of this study was to evaluate trunk muscle function, including respiratory muscles, in patients with MD2 and to compare it with healthy controls, to determine the occurrence of CLBP in patients with MD2, and to assess whether trunk muscle dysfunction increases the risk of CLBP in these patients.

**Methods:**

We enrolled 40 MD2 patients (age range 23 to 76 years, 26 women). A comprehensive battery of tests was used to evaluate trunk muscle function. The tests consisted of quantitative muscle strength testing of low back extensor muscles and respiratory muscles and the assessment of trunk muscle endurance. A neurological evaluation contained procedures assessing the distribution of muscle weakness, myotonia, and pain, and used questionnaires focused on these items and on disability, depression, and physical activity.

**Results:**

The results of this study suggest that patients with MD2 show significant dysfunction of the trunk muscles, including the respiratory muscles, expressed by decreased muscle strength and endurance. The prevalence of CLBP in patients with MD2 was 52.5%. Based on our analysis, the only independent significant risk factor for CLBP in these patients was maximal isometric lower back extensor strength in a prone position ≤ 15.8 kg (OR = 37.3). Other possible risk factors were severity of myotonia and reduced physical activity.

**Conclusion:**

Outcomes of this study highlighted the presence of axial muscle dysfunction, respiratory muscle weakness, and frequent occurrence of CLBP together with its risk factors in patients with MD2. We believe that the findings of this study may help in management and prevention programs for patients with MD2.

## 1. Introduction

Myotonic dystrophy type 2 (MD2) is the most common adult-onset muscular dystrophy in Central Europe and Northern Europe (Finland); the estimated prevalence of MD2 is about 9 in 100.000 (1). It is typified by a varied manifestation such as early onset cataracts, various grip myotonias, stiffness of thigh muscle, muscular pain, and weakness (2, 3). Patients with MD2 reveal the weakness of proximal muscles of limbs and truncal (axial) muscles. Later in the disease course, muscular weakness advances from proximal to distal muscles (4). Respiratory muscle weakness is a typical feature of myotonic dystrophy type 1 (MD1) and contributes to respiratory dysfunction and failure. On the other hand, respiratory complications and failure are thought to be uncommon in MD2 (2, 5, 6). Pain is a common and very serious problem for many MD2 patients, with a lifetime prevalence estimated at 76% and a negative impact on quality of life (7, 8). Pain most often occurs symmetrically in the proximal limbs and lower back (2, 7). It is recommended to supply patients with a rehabilitation plan, individualized in terms of the appropriate type and intensity of exercise, which should be done by a consulting physical therapist (4).

**Figure 1:**
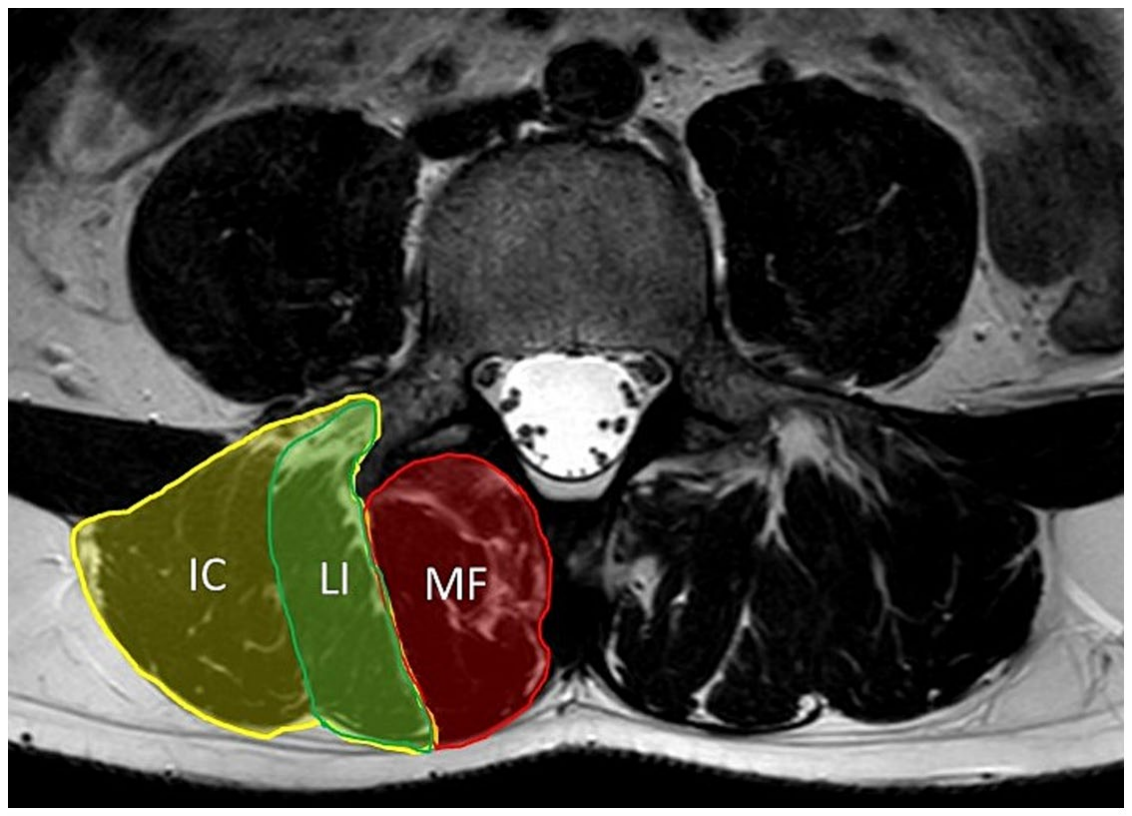
Axial T2-weighted MRI of lumbar spine at level of L4. Figure shows anatomy of lumbar paraspinal muscles. The erector spinae muscle consists of the longissimus (LI) and iliocostalis (IC) muscles. The multifidus muscle (MF) is medial to the erector spinae muscle.

Many recognized myopathies have significant affection of the axial musculature. This is also described in patients with both types of myotonic dystrophy, classified as axial myopathies with prominent paraspinal involvement as part of a more widespread myopathy (9). Axial muscles are a component of the deep spinal (core) stabilization system, which consists of the lumbar paraspinal muscles (particularly the erector spinae muscle and the lumbar multifidus muscle that are part of the lumbar extensor muscle system as shown in Figure 1), abdominal muscles, diaphragm, and pelvic floor muscles. Paraspinal muscles are extremely important as they help to maintain proper posture and protect the spinal segments (10, 11). It has been reported that dysfunction of the deep spinal stabilization system, causing trunk instability, may lead to chronic low back pain (CLBP) over time, which is a common condition in the general population (12). Laroche et al. reported that up to 66% of patients with axial myopathy experience back pain (13).

Although trunk muscles have a very important function in posture and mobility and probably play a significant role in spinal health, their comprehensive examination is often neglected. In our previous study, we defined a battery of tests that comprehensively assess trunk muscle function (including muscle strength, muscle endurance, and coordination) and have the potential to reveal trunk muscle dysfunction (14, 15).

The purpose of this study was to evaluate trunk muscle function including respiratory muscles in patients with MD2 and to compare it with healthy controls, to determine the occurrence of CLBP in patients with MD2, and to assess whether trunk muscle dysfunction increases the risk of CLBP in these patients. The hypothesis was that patients with MD2 exhibit trunk muscle dysfunction that contributes to the high incidence of CLBP in these patients. We expect that trunk muscle evaluation in patients with certain neuromuscular diseases, including MD2, will allow better monitoring of muscle damage and dysfunction, help to explain the frequent occurrence of low back pain, and facilitate the targeting of rehabilitation programs for these patients.

## 2. Materials and methods

This was a prospective cross-sectional observational comparative study. The local institutional medical research ethics committee approved the study protocol (agreement number 05-090621/EK), and written informed consent was obtained from all participants.

### 2.1. Participants

#### 2.1.1. Patients with MD2

Patients with MD2 were prospectively recruited from a cohort of patients with a confirmed diagnosis of MD2 who were evaluated at the Neuromuscular Center of the University Hospital, were enrolled in the Registry of muscular dystrophies REaDY, and had a neurological check-up between May 2021 and March 2023.

The inclusion criteria were MD2 confirmed by molecular genetic testing and age > 18 years. Exclusion criteria were limiting paresis evaluated by manual muscle testing of the hip extension and defined by the Medical Research Council (MRC) scale less than grade 4-, previous vertebral fracture, spine infection or tumor of the lumbar spine, comorbid conditions affecting the overall mobility of the patient (e.g., poststroke paresis, heart failure leading to limited mobility), confirmed pregnancy, and significant impairment of cognitive functions.

#### 2.1.2. Healthy volunteers

Healthy volunteers (HV) were recruited from a control database of 115 individuals that was developed for a long-term project investigating the function and morphology of the lumbar paraspinal muscles at our neuromuscular center. Healthy controls were obtained from two sources. Half of the healthy controls were employees of the University Hospital, including medical doctors, physiotherapists, nurses, and hospital support staff. Personal recruitment by experienced staff (recruitment agency) was used to gather the second half of volunteers from the population of the province of South Moravia (south-eastern Czech Republic). The guidelines included an established quota of subjects intended to obtain a proportional representation of four age categories (18–29, 30–39, 40–49, 50–65) and both sexes.

Exclusion criteria were: age < 18 years, acute low back pain, a medical history of chronic low back pain (of duration over 12 weeks), presence of lumbosacral radicular pain in the medical record with residual signs of nerve root dysfunction (sensory impairment and/or absent or diminished reflexes and/or weakness) in clinical neurological examination and manual muscle testing of the lower extremities, previous surgery of the lumbar spine, vertebral fracture, spine infection or tumor, presence of myopathy, comorbid conditions affecting the overall mobility of the patient (e.g., post-stroke paresis, heart failure leading to limited mobility), and confirmed pregnancy. To enable the comparison of a group of MD2 patients with HV, a subgroup of HV was selected from the whole HV database. We used the optimal pair propensity score matching to assign the patients with MD2 to healthy volunteers with similar sex, age, and body mass index (BMI).

### 2.2. Procedures

All subjects underwent a detailed neurological clinical evaluation, including a medical history taken by an expert neurologist and a functional motor assessment of the trunk muscles by a physiotherapist.

#### 2.2.1. Medical history and neurological clinical evaluation

The medical histories of HV and patients with MD2 were taken to evaluate the presence of exclusion criteria and comorbidities. In the patients with MD2, we further focused on the presence of diabetes mellitus, the presence of chronic musculoskeletal pain (except low back pain), and smoking. The presence of low back pain (LBP) was assessed and, if present, other parameters were evaluated, specifically whether the pain was acute or chronic (lasting more than 12 weeks), the exact duration of the pain (in weeks), and axial (non-specific) pain or radicular syndrome. Radicular syndrome was defined as typical dermatomal pain radiating beyond the knee toward the foot, together with at least one sign of nerve root dysfunction: sensory impairment and/or absent or diminished reflexes and/or weakness. LBP was also closely analyzed regarding intensity. Pain intensity was measured using an 11-point numerical rating scale (NRS: 0–10). Current pain intensity was recorded along with the average and maximum pain intensity over the previous 4 weeks. Patients were asked about the use of analgesic medication or myorelaxants as well as whether they underwent manual or exercise therapy for LBP. LBP-related disability was assessed using the Oswestry Disability Index (ODI) (range 0–100%) (16, 17) and the Roland-Morris Disability Questionnaire (RMQ) (range 0–24) (18). Symptoms of depression were evaluated with the Beck Depression Inventory II (BDI II) which has shown to be a suitable psychometric tool capable of quantifying the severity of depression (range 0–63) (19, 20). The short form of the International Physical Activity Questionnaire (IPAQ) was used to evaluate the amount of daily physical activity performed by every subject. It has three questions assessing levels of physical activities. Each of these was to be answered regarding frequency of activities per week and time spent doing the activity per session. These data have been applied to produce a result expressed as a total metabolic equivalent (MET) in minutes per week (METs are multiples of the resting metabolic rate).

All subjects also underwent a comprehensive neurological examination to evaluate the possible presence of lumbosacral radicular dysfunction and clinical symptoms of myopathy. To evaluate the muscle strength of lower limbs and its girdle we used a manual muscle test rating with the MRC scale. Nine muscle groups with respect to their physiological function in a certain joint were assessed in each subject. Then a sum of all 18 scores was calculated to get the overall strength of lower limbs (range 0–90). The muscle strength of lumbar extensors was examined using a manual muscle test graded with the MRC scale (range 0–5).

The clinical presence of myotonia in patients with MD2 was assessed, specifically percussion myotonia by tapping on the thumb abductor muscle and grip myotonia. The assessment of myotonia severity was evaluated with a specific questionnaire: the Myotonia Behavior Scale (range 0–5) (21, 22).

#### 2.2.2. Functional assessment of muscle trunk

To assess trunk muscle function in more detail, a battery of simple tests including strength and endurance measurements specific for core muscles with an emphasis on examining the back extensors, including the lumbar paraspinal muscles, was used. The muscle strength testing procedures consist of measurement of maximal isometric lower back extensor strength using a handheld dynamometer MicroFET 2 (Hoggan Scientific, LLC.) in three postural positions (prone, sitting, and standing) and measurement of respiratory muscle strength—maximal inspiratory muscle strength (maximum inspiratory pressure, MIP) and maximal expiratory muscle strength (maximum expiratory pressure, MEP)—using the microRPM (Micro Medical, Kent, United Kingdom) electronic pressure gauge. The Biering-Sørensen test was used to assess trunk and hip extensor endurance. Further tests challenged the endurance of the core muscles and those were the prone-plank test for the abdominal core muscles and the side-bridge test (on both sides) for the lateral core muscles.

The methodology is described in detail in a previous work by some of the authors of this study (14).

### 2.3. Statistical approaches

Descriptive statistical analysis respected the type of data and the distribution of the values. Continuous parameters were described using median with minimum (min) and maximum (max). The summary of the categorical parameters was done using absolute and relative frequencies. Relative frequencies were calculated using the number of individuals in the relevant subgroup. In accordance with the data type (categorical × continuous), Pearson’s chi-square (resp. Fisher’s exact test in case of non-meeting criteria), or the Mann–Whitney *U*-test (also known as the Wilcoxon rank-sum test) were used to examine the association between selected variables. After matching, McNemar’s chi-squared test (paired) and the Wilcoxon signed rank test (paired) were used to examine the association between selected variables.

The diagnostic power of potential risk factors of CLBP in patients with MD2 was assessed by means of receiver operating characteristic (ROC) curves and described using area under the curve (AUC) and its statistical significance (tested with the Wilcoxon rank-sum test); optimal cut-offs were identified using a combination of their sensitivity and specificity. We used logistic regression to determine risk factors for the occurrence of CLBP, so this variable was used as a response. All variables that were considered potential risk factors were used in one-dimensional logistic regression. The variables with value of p in ROC analysis < 0.1 were categorized based on their optimal cut-off point and then used in one-dimensional logistic regression in both (continuous and binary) forms. Next, we wanted to explain CLBP using multidimensional logistic regression. All variables with value of p from one-dimensional logistic regression models < 0.1 were used. If a variable in one-dimensional logistic regression was significant in both forms, as continuous and binary, the binary type was used. The best model was chosen using forward stepwise selection. All statistical tests were performed at a significance level of α = 0.05 (all tests were two-sided). Statistical analyses were performed using R software (version 4.1.2) (23).

## 3. Results

### 3.1. Basic characteristics of participants

**Figure 2:**
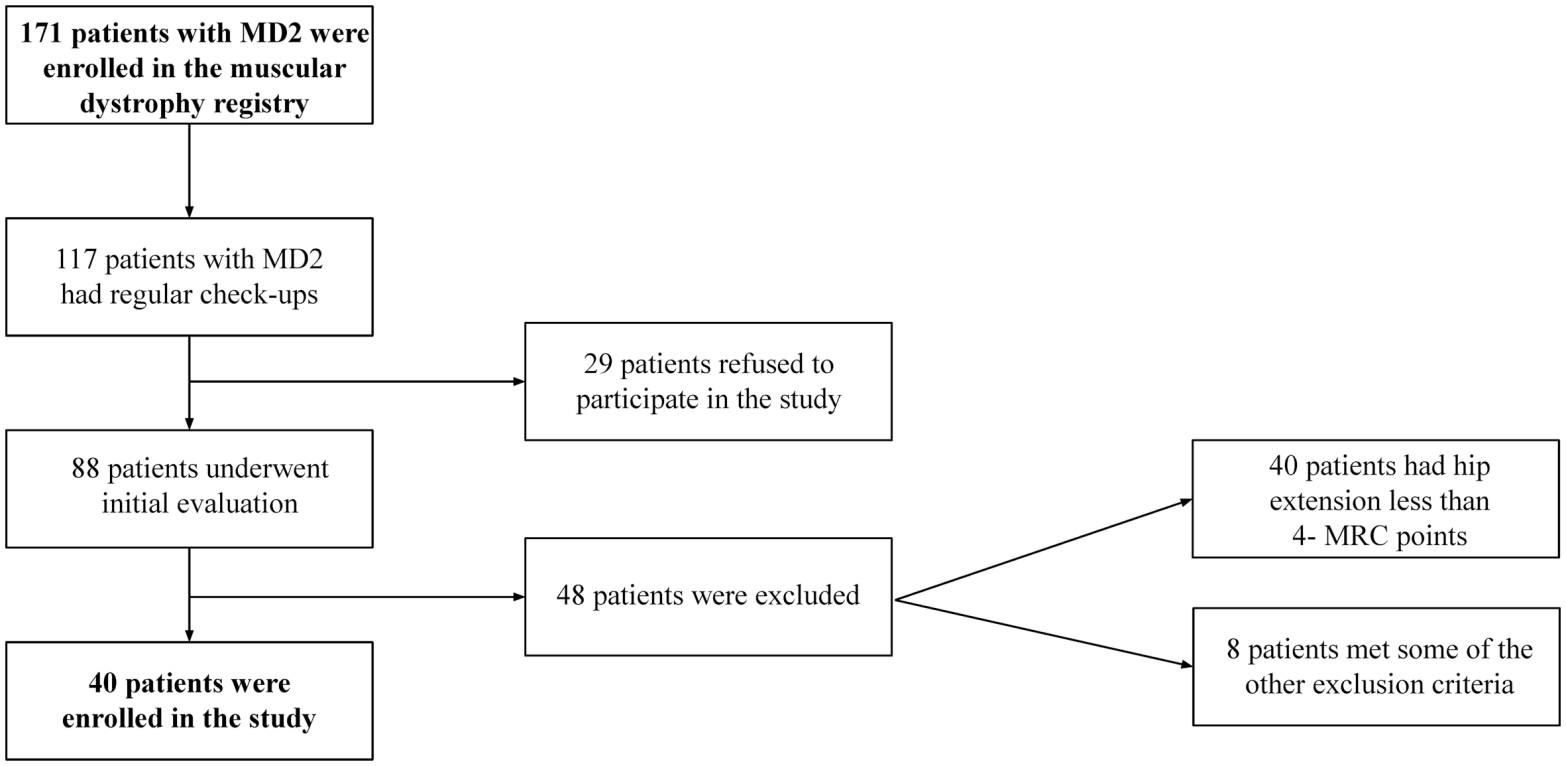
Recruitment flowchart for patients with myotonic dystrophy type 2.

The basic characteristics (age, sex, and BMI) of patients with MD2 (*n* = 40; consisting of 38 different MD2 families) and the whole group of HV (*n* = 115) appear in Table 1 (data are compiled from all HV). Figure 2 summarizes the recruitment of patients with MD2. A statistically significant difference between HV and patients with MD2 was found in age and BMI. For this reason, we matched the patients with MD2 with HV based on their age, BMI, and sex; we formed two groups of 40 individuals. The basic characteristics are presented in Table 2. Regarding the functional status of patients with MD2, 26 were able to stand up from a standardized 40 cm high chair without any compensatory maneuver, 7 patients used a compensatory maneuver, 4 used one hand and 3 used two hands to help them stand up. All of them but one did not use any walking aid. Patients with MD2 significantly differed in education and physical activity; they had a lower level of education and lower physical activity expressed in MET (minutes per week). Further analyses were based on data from the matching groups of patients with MD2 and HV.

**Table 1:** Basic characteristics of patients with MD2 and healthy volunteers before matching.

	Healthy volunteers	Myotonic dystrophy type 2	*p*-value^1^
	*N* = 115	*N* = 40	
Age (years)			**<0.001**
Median (min; max)	37.0 (20.0; 78.0)	50.5 (23.0; 76.0)	
BMI (kg/m^2^)			**0.022**
Median (min; max)	23.6 (17.9; 37.0)	25.4 (16.9; 39.7)	
Sex, *n* (%)			0.111
Female	58 (50.4)	26 (65.0)	
Male	57 (49.6)	14 (35.0)	

^1^Wilcoxon rank-sum test, Pearson’s chi-squared test. BMI, body mass index; MD2, myotonic dystrophy type 2; *N*, number of individuals.

The bold values are statistically significant (significance level of *α* = 0.05).

**Table 2:** Basic characteristics of patients with MD2 and healthy volunteers after matching.

	Healthy volunteers	Myotonic dystrophy type 2	*p*-value^1^
	*N* = 40	*N* = 40	
Sex, *n* (%)			>0.999
Female	25 (62.5)	26 (65.0)	
Male	15 (37.5)	14 (35.0)	
Age (years)			0.326
Median (min; max)	48.0 (25.0; 78.0)	50.5 (23.0; 76.0)	
BMI (kg/m^2^)			0.476
Median (min; max)	24.6 (20.3; 37.0)	25.4 (16.9; 39.7)	
Education, *n* (%)			**<0.001**
Basic	1 (2.5)	16 (40.0)	
Secondary	17 (42.5)	21 (52.5)	
Tertiary	22 (55.0)	3 (7.5)	
MET (minutes per week)			**0.007**
Median (min; max)	2,025.5 (0.0; 7,119.0)	1,276.0 (0.0; 5,544.0)	

^1^McNemar’s Chi-squared test (paired), Wilcoxon signed rank test (paired). BMI, body mass index; MD2, myotonic dystrophy type 2; MET, multiples of the resting metabolic rate; *N*, number of individuals. Basic education, graduated from elementary school; Secondary education, graduated from secondary school with state exam; Tertiary education, graduated from university.

The bold values are statistically significant (significance level of *α* = 0.05).

### 3.2. Assessment of trunk muscle function

We found a very highly statistically significant difference (value of *p* < 0.001) between healthy volunteers and patients with MD2 in all examined functional parameters of trunk muscles (Table 3). Patients with MD2 demonstrated significantly reduced maximal isometric lower back extensor strength in all positions (prone, sitting, and standing), decreased strength of both inspiratory and expiratory muscles (evaluated by maximum inspiratory and expiratory pressures), and considerably worse results in trunk muscle endurance tests (Biering-Sørensen test, prone-plank test, side-bridge test for both sides). Some patients were unable to perform the endurance test due to trunk muscle impairment, so they scored zero seconds; this did not occur in any healthy volunteers.

**Table 3:** Functional examination of trunk muscles.

	Healthy volunteers	Myotonic dystrophy type 2	*p*-value^1^
	*N* = 40	*N* = 40	
Maximal isometric lower back extensor strength—prone position (kg)^†^			**<0.001**
Median (min; max)	24.0 (15.7; 38.7)	18.0 (4.7; 32.4)	
Maximal isometric lower back extensor strength—sitting position (kg)^†^			**<0.001**
Median (min; max)	45.7 (17.9; 100.6)	27.2 (5.9; 82.7)	
Maximal isometric lower back extensor strength—standing position (kg)^†^			**<0.001**
Median (min; max)	40.5 (18.4; 78.2)	27.9 (5.5; 76.0)	
Respiratory muscle strength—maximum inspiratory pressure (cm H_2_O)^††^			**<0.001**
Median (min; max)	83.8 (38.0; 141.3)	56.7 (14.7; 137.7)	
Respiratory muscle strength—maximum expiratory pressure (cm H_2_O)^††^			**<0.001**
Median (min; max)	120.3 (66.7; 217.3)	88.3 (57.3; 147.7)	
Biering-Sørensen test [time(s)]			**<0.001**
Median (min; max)	150.5 (24.0; 307.0)	47.5 (0.0; 298.0)	
Prone-plank test [time(s)]			**<0.001**
Median (min; max)	120.5 (22.0; 207.0)	54.5 (0.0; 174.0)	
Side-bridge test—right side [time(s)]			**<0.001**
Median (min; max)	52.5 (12.0; 121.0)	9.5 (0.0; 65.0)	
Side-bridge test—left side [time (s)]			**<0.001**
Median (min; max)	54.5 (15.0; 121.0)	8.5 (0.0; 78.0)	

^1^Wilcoxon signed rank test (paired). ^†^Maximal isometric lower back extensor strength is calculated from the mean value of attempt 2 to attempt 5. ^††^Respiratory muscle strength is calculated from the mean value of attempt 2 to attempt 4. N: number of individuals.

The bold values are statistically significant (significance level of *α* = 0.05).

### 3.3. Chronic low back pain in patients with MD2

Patients with MD2 were divided into two subgroups with respect to the presence of chronic low back pain. No patient had acute LBP. The total number of patients with CLBP was 21 (52.5% of 40 patients with MD2), and the description of CLBP attribute is in Table 4. The median duration of CLBP was 520 weeks and the median average pain intensity in the previous 4 weeks was 4.0. Patients with axial CLBP (66.7%) exceeded those with radicular pain. Patients with CLBP were often using analgesic medication for CLBP (76.2%), most frequently non-steroidal anti-inflammatory drugs (NSAIDs), only one patient was using a central myorelaxant. A total of 10 patients with MD2 and CLBP were currently undergoing rehabilitation procedures, most often a combination of manual and exercise therapy (seven patients); two patients were undergoing manual therapy only, and one patient was undergoing exercise therapy only. Disability in relation to CLBP in patients with MD2, assessed by the questionnaires, was predominantly mild, median Oswestry Disability Index (ODI) was 17.8% and the median Roland-Morris Disability Questionnaire (RMQ) score was 6.0 points.

**Table 4:** Attributes of CLBP for patients with MD2.

Chronic low back pain—patients with myotonic dystrophy type 2	*N* = 21
Duration of CLBP (weeks)	
Median (min; max)	520.0 (12.0; 1,924.0)
Axial chronic low back pain, *n* (%)	14 (66.7)
Radicular chronic low back pain, *n* (%)	7 (33.3)
Average CLBP intensity (in the previous 4 weeks) (NRS 0–10)	
Median (min; max)	4.0 (1.0; 7.0)
Maximum CLBP intensity (NRS 0–10)	
Median (min; max)	6.0 (2.0; 10.0)
Current CLBP intensity (NRS 0–10)	
Median (min; max)	2.0 (0.0; 6.0)
Use of analgesic medication for CLBP, *n* (%)	16 (76.2)
Use of central myorelaxants, *n* (%)	1 (4.8)
Ongoing manual therapy, *n* (%)	9 (42.9)
Ongoing exercise therapy, *n* (%)	8 (38.1)
Radicular lumbosacral paresis, *n* (%)	1 (4.8)
Radicular lumbosacral sensory impairment, *n* (%)	2 (9.5)
Oswestry disability index (0%–100%)	
Median (min; max)	17.8 (4.4; 33.0)
Roland-Morris Disability Questionnaire (0–24)	
Median (min; max)	6.0 (1.0; 18.0)

CLBP, Chronic low back pain; MD2, myotonic dystrophy type 2; *N*, number of individuals; NRS, Numerical Rating Scale.

### 3.4. Predictors of chronic low back pain in patients with MD2

In order to predict the occurrence of CLBP in patients with MD2, we evaluated the difference in individual parameters between subgroups of MD2 patients with and without CLBP (Table 5). We found no difference between the two subgroups of MD2 patients in the demographic parameters (sex, age), they did not differ in BMI, education level, smoking, the prevalence of comorbidities (diabetes mellitus or impaired glucose tolerance, the presence of musculoskeletal pain except LBP, depression), severity of myotonia as assessed by Myotonia Behavior Scale, or strength of lumbar extensor muscles as assessed by the MRC scale. However, the two subgroups of MD2 differed statistically significantly in some parameters of trunk muscle function, namely in maximal isometric lower back extensor strength in all positions (assessed by handheld dynamometer) and in the Biering-Sørensen test. There was a borderline significant difference in side-bridge test performance (for the test on the right side, the difference was statistically significant) as well as in amount of physical activity (MET), clinical presence of myotonia, and summary strength of lower limbs as assessed by the MRC scale. There was no statistically significant difference in respiratory muscle strength (both inspiratory and expiratory) or in the prone-plank test, although MD2 patients with CLBP performed worse on these tests than patients without CLBP.

**Table 5:** Comparing subgroups of patients with MD2 (with and without CLBP).

	Total	Without CLBP	With CLBP	*p*-value^1^
	*N* = 40	*N* = 19	*N* = 21	
Age (years)				0.989
Median (min; max)	50.5 (23.0; 76.0)	51.0 (25.0; 67.0)	50.0 (23.0; 76.0)	
Sex, *n* (%)				0.816
Female	26 (65.0)	12 (63.2)	14 (66.7)	
Male	14 (35.0)	7 (36.8)	7 (33.3)	
BMI (kg/m^2^)				0.810
Median (min; max)	25.4 (16.9; 39.7)	24.8 (16.9; 39.7)	26.1 (20.3; 34.0)	
Education, *n* (%)				0.712
Basic	16 (40.0)	8 (42.1)	8 (38.1)	
Secondary	21 (52.5)	9 (47.4)	12 (57.1)	
Tertiary	3 (7.5)	2 (10.5)	1 (4.8)	
Smoking, *n* (%)	4 (10.0)	3 (15.8)	1 (4.8)	0.331
Diabetes mellitus or impaired glucose tolerance, *n* (%)	5 (12.5)	3 (15.8)	2 (9.5)	0.654
Chronic musculoskeletal pain except low back pain [pain intensity at least 4 (NRS)], *n* (%)	21 (52.5)	10 (52.6)	11 (52.4)	0.987
Presence of myotonia, *n* (%)	19 (47.5)	6 (31.6)	13 (61.9)	0.055
Myotonia Behavior Scale (0–5), *n* (%)				0.104
0	8 (20.0)	6 (31.6)	2 (9.5)	
1	16 (40.0)	9 (47.4)	7 (33.3)	
2	12 (30.0)	3 (15.8)	9 (42.9)	
3	4 (10.0)	1 (5.3)	3 (14.3)	
4	0 (0.0)	0 (0.0)	0 (0.0)	
5	0 (0.0)	0 (0.0)	0 (0.0)	
MET (minutes per week)				0.078
Median (min; max)	1,276.0 (0.0; 5,544.0)	1,455.0 (198.0; 4,158.0)	1,020.0 (0.0; 5,544.0)	
Beck Depression Inventory II (0–63)				0.385
Median (min; max)	7.0 (0.0; 35.0)	6.0 (0.0; 35.0)	8.0 (0.0; 31.0)	
Strength of lumbar extensor muscles (0–5), *n* (%)				0.163
0	0 (0.0)	0 (0.0)	0 (0.0)	
1	0 (0.0)	0 (0.0)	0 (0.0)	
2	9 (22.5)	2 (10.5)	7 (33.3)	
3	7 (17.5)	2 (10.5)	5 (23.8)	
4	15 (37.5)	9 (47.4)	6 (28.6)	
5	9 (22.5)	6 (31.6)	3 (14.3)	
Maximal isometric lower back extensor strength—prone position (kg)^†^				**0.008**
Median (min; max)	18.0 (4.7; 32.4)	19.4 (13.8; 32.4)	15.7 (4.7; 28.0)	
Maximal isometric lower back extensor strength—sitting position (kg)^†^				**0.044**
Median (min; max)	27.2 (5.9; 82.7)	30.6 (17.1; 82.7)	24.4 (5.9; 67.1)	
Maximal isometric lower back extensor strength—standing position (kg)^†^				**0.032**
Median (min; max)	27.9 (5.5; 76.0)	31.2 (14.2; 76.0)	22.1 (5.5; 55.4)	
Respiratory muscle strength—maximum inspiratory pressure (cm H_2_O)^††^				0.273
Median (min; max)	56.7 (14.7; 137.7)	58.7 (33.3; 98.3)	53.3 (14.7; 137.7)	
Respiratory muscle strength—maximum expiratory pressure (cm H_2_O)^††^				0.343
Median (min; max)	88.3 (57.3; 147.7)	88.7 (64.3; 147.7)	88.0 (57.3; 139.3)	
Biering-Sørensen test [time(s)]				**0.012**
Median (min; max)	47.5 (0.0; 298.0)	103.0 (4.0; 298.0)	35.0 (0.0; 123.0)	
Prone-plank test [time(s)]				0.155
Median (min; max)	54.5 (0.0; 174.0)	59.0 (6.0; 174.0)	33.0 (0.0; 129.0)	
Side-bridge test—right side [time(s)]				**0.046**
Median (min; max)	9.5 (0.0; 65.0)	20.0 (0.0; 65.0)	6.0 (0.0; 61.0)	
Side-bridge test—left side [time (s)]				0.074
Median (min; max)	8.5 (0.0; 78.0)	22.0 (0.0; 78.0)	4.0 (0.0; 61.0)	
Summary strength of lower limbs (MRC) (0–90)				0.075
Median (min; max)	83.2 (70.5; 90.0)	85.0 (77.5; 90.0)	81.0 (70.5; 90.0)	

^1^Wilcoxon rank-sum test, Pearson’s chi-squared test, Fisher’s exact test. ^†^Maximal isometric lower back extensor strength is calculated from the mean value of attempt 2 to attempt 5. ^††^Respiratory muscle strength is calculated from the mean value of attempt 2 to attempt 4. BMI, body mass index; CLBP, Chronic low back pain; MD2, myotonic dystrophy type 2; MET, multiples of the resting metabolic rate; MRC, Medical Research Council Scale for Muscle Strength (0–5); N, number of individuals; NRS, Numerical Rating Scale. Basic education, graduated from elementary school; Secondary education, graduated from secondary school with state exam; Tertiary education, graduated from university.

The bold values are statistically significant (significance level of *α* = 0.05).

ROC analysis of potential risk factors for CLBP in patients with MD2 disclosed the following parameters as effective discriminating factors between the two subgroups of patients: Myotonia Behavior Scale ≥ 2, the strength of lumbar extensor muscles (by the MRC scale) ≤ 3, maximal isometric lower back extensor strength—prone position ≤ 15.8 kg, maximal isometric lower back extensor strength—sitting position ≤ 37.5 kg, maximal isometric lower back extensor strength—standing position ≤ 29.0 kg, Biering-Sørensen test ≤ 123 s and side-bridge test—right side ≤ 8 s (Table 6).

**Table 6:** ROC analysis of potential risk factors of CLBP in patients with MD2.

Variable	Cut-point	AUC	*p*-value^1^	Sensitivity	Specificity
Age (years)	≥71	0.503	0.989	0.095	1.000
BMI (kg/m^2^)	≥23.5	0.524	0.807	0.714	0.474
Chronic musculoskeletal pain except low back pain—average pain intensity (in the previous 4 weeks)	≥7	0.505	>0.999	0.091	1.000
Myotonia Behavior Scale (0–5)	≥2	0.711	**0.017**	0.571	0.789
MET (minutes per week)	≤1,272	0.664	0.078	0.667	0.684
Beck Depression Inventory II (0–63)	≥7	0.581	0.385	0.667	0.579
Strength of lumbar extensor muscles (0–5)	≤3	0.697	**0.028**	0.571	0.789
Maximal isometric lower back extensor strength—prone position (kg)^†^	≤15.8	0.744	**0.009**	0.571	0.947
Maximal isometric lower back extensor strength—sitting position (kg)^†^	≤37.5	0.687	**0.045**	0.857	0.474
Maximal isometric lower back extensor strength—standing position (kg) ^†^	≤29.0	0.699	**0.032**	0.714	0.632
Respiratory muscle strength—maximum inspiratory pressure (cm H_2_O) ^††^	≤71.0	0.603	0.273	0.857	0.368
Respiratory muscle strength—maximum expiratory pressure (cm H_2_O) ^††^	≤113.3	0.589	0.343	0.810	0.368
Biering-Sørensen test [time(s)]	≤123	0.733	**0.012**	1.000	0.474
Prone-plank test [time(s)]	≤9	0.633	0.155	0.381	0.947
Side-bridge test—right side [time(s)]	≤8	0.684	**0.046**	0.619	0.737
Side-bridge test—left side [time (s)]	≤7	0.664	0.074	0.667	0.684
Summary strength of lower limbs (MRC) (0–90)	≤82.5	0.665	0.075	0.667	0.684

^1^Wilcoxon rank-sum test. ^†^Maximal isometric lower back extensor strength is calculated from the mean value of attempt 2 to attempt 5. ^††^Respiratory muscle strength is calculated from the mean value of attempt 2 to attempt 4. AUC, area under the curve; BMI, body mass index; CLBP, Chronic low back pain; MD2, myotonic dystrophy type 2; MET, multiples of the resting metabolic rate; MRC, Medical Research Council Scale for Muscle Strength (0–5).

The bold values are statistically significant (significance level of *α* = 0.05).

One-dimensional logistic regression led to statistically significant OR [crude OR (Odds ratio)] for the occurrence of CLBP in patients with MD2 at these parameters (Table 7): Myotonia Behavior Scale ≥ 2 (OR = 5.0), MET ≤ 1,272 min per week (OR = 4.3), the strength of lumbar extensor muscles (by the MRC scale) ≤ 3 (OR = 5.0), maximal isometric lower back extensor strength—prone position ≤ 15.8 kg (OR = 24.0), maximal isometric lower back extensor strength—sitting position ≤ 37.5 kg (OR = 5.4), maximal isometric lower back extensor strength—standing position ≤ 29.0 kg (OR = 4.3), prone-plank test ≤ 9 s (OR = 11.1), side-bridge test—right side ≤ 8 s (OR = 4.6), side-bridge test—left side ≤ 7 s (OR = 4.3), summary strength of lower limbs ≤ 82.5 (OR = 4.3).

**Table 7:** Potential risk factors of CLBP in patients with MD2 in logistic regression models.

		One-dimensional			Multidimensional^2^		
Variable	% with CLBP^1^	OR	95% CI	*p*-value^3^	OR	95% CI	*p*-value^3^
Age (years)	-	1.01	0.96–1.06	0.826			
Height (cm)	-	1.04	0.97–1.13	0.313			
Weight (kg)	-	1.00	0.97–1.04	0.886			
BMI (kg/m^2^)	-	0.98	0.87–1.10	0.720			
Sex: Male	50.0	0.86	0.23–3.19	0.816			
Diabetes mellitus or impaired glucose tolerance: Yes	40.0	0.56	0.07–3.79	0.553			
Smoking: Yes	25.0	0.27	0.01–2.31	0.272			
Chronic musculoskeletal pain except low back pain [pain intensity at least 4 (NRS)]: Yes	52.4	0.99	0.28–3.46	0.987			
Presence of myotonia: Yes	68.4	3.52	0.98–13.81	0.059			
Myotonia Behavior Scale	-	2.55	1.19–6.39	**0.026**			
Myotonia Behavior Scale: ≥2	75.0	5.00	1.31–22.54	**0.024**	6.18	1.06–52.35	0.057
MET (minutes per week)	-	1.00	1.00–1.00	0.312			
MET: ≤1,272	70.0	4.33	1.20–17.43	**0.030**	5.80	1.06–47.83	0.060
Beck Depression Inventory II (0–63)	-	1.03	0.96–1.11	0.485			
Strength of lumbar extensor muscles (0–5)	-	0.48	0.23–0.90	**0.031**			
Strength of lumbar extensor muscles (0–5): ≤3	75.0	5.00	1.31–22.54	**0.024**			
Maximal isometric lower back extensor strength—prone position (kg)^†^	-	0.84	0.72–0.95	**0.011**			
Maximal isometric lower back extensor strength—prone position (kg): ≤15.8^†^	92.3	24.00	3.84–473.57	**0.004**	37.25	4.25–985.97	**0.006**
Maximal isometric lower back extensor strength—sitting position (kg)^†^	-	0.97	0.93–1.00	0.077			
Maximal isometric lower back extensor strength—sitting position (kg) ≤ 37.5^†^	64.3	5.40	1.28–28.85	**0.029**			
Maximal isometric lower back extensor strength—standing position (kg)^†^	-	0.95	0.90–0.99	**0.041**			
Maximal isometric lower back extensor strength—standing position (kg): ≤29.0^†^	68.2	4.29	1.18–17.27	**0.032**			
Respiratory muscle strength—maximum inspiratory pressure (cm H_2_O)^††^	-	0.99	0.96–1.02	0.414			
Respiratory muscle strength—maximum expiratory pressure (cm H_2_O)^††^	-	0.99	0.96–1.01	0.299			
Biering-Sørensen test [time(s)]	-	0.98	0.97–0.99	**0.009**			
Biering-Sørensen test [time(s)]: ≤123	67.7	-	-	0.993			
Prone-plank test [time(s)]	-	0.99	0.98–1.00	0.158			
Prone-plank test [time(s)]: ≤9	88.9	11.08	1.73–218.86	**0.032**			
Side-bridge test—right side [time(s)]	-	0.97	0.94–1.00	0.084			
Side-bridge test—right side [time(s)]: ≤8	72.2	4.55	1.24–18.93	**0.028**			
Side-bridge test—left side [time (s)]	-	0.98	0.95–1.00	0.107			
Side-bridge test—left side [time (s)]: ≤7	70.0	4.33	1.20–17.43	**0.030**			
Summary strength of lower limbs (MRC) (0–90)	-	0.88	0.77–0.99	**0.048**			
Summary strength of lower limbs (MRC) (0–90): ≤82.5	70.0	4.33	1.20–17.43	**0.030**			

^1^Percentage of patients with CLBP within a category given by the risk factor (only for categorized predictors). ^2^Only statistically significant multivariate-adjusted OR is displayed (variables selected by forward stepwise selection). ^3^Wald test. ^†^Maximal isometric lower back extensor strength is calculated from the mean value of attempt 2 to attempt 5. ^††^Respiratory muscle strength is calculated from the mean value of attempt 2 to attempt 4. BMI, body mass index; CLBP, Chronic low back pain; MD2, myotonic dystrophy type 2; MET, multiples of the resting metabolic rate; MRC, Medical Research Council Scale for Muscle Strength (0–5); NRS, Numerical Rating Scale; OR, Odds ratio; 95% CI: 95% confidence interval.

The bold values are statistically significant (significance level of *α* = 0.05).

Multidimensional logistic regression proposed only maximal isometric lower back extensor strength—prone position ≤ 15.8 kg (OR = 37.3, *p* = 0.006) as an independent risk factor for CLBP in patients with MD2. The other two parameters, namely Myotonia Behavior Scale score ≥ 2 (OR = 6.2) and MET ≤ 1,272 min per week (OR = 5.8), had borderline statistical significance as independent risk factors for CLBP in patients with MD2 (Table 7).

## 4. Discussion

This study focused on analyzing the trunk muscle function, including respiratory muscles, in patients with MD2. At the same time, it evaluated the occurrence of CLBP in these patients and identified the risk factors for this comorbidity. The hypothesis of the study has been proven. The results suggest that patients with MD2 show significant dysfunction of the trunk muscles, including respiratory muscles, and thus have an impaired deep spinal stabilization system. Decreased muscle strength of the lower back extensors together with a possible contribution of myotonia severity and reduced physical activity appear to be risk factors for the frequent occurrence of CLBP in patients with MD2.

The literature describing functional examinations of axial myopathy and particular assessment methods for the paraspinal musculature is sparse. In the field of neuromuscular disorders, motor function scales such as the Hammersmith Functional Motor Scale and the Motor Function Measure are used most frequently (9, 24, 25). In terms of functional tests used in MD2, the most common tools are the Gait, Stairs, Gower, Chair (GSGC) scale, the 30 s sit-to-stand test, and the 6-min walking test (26). However, those estimate complex functions rather than specific muscles; in the context of axial myopathy, those scales are too unspecific. Observations of spine mobility and manual muscle testing (MMT) are very basic and commonly used diagnostic tools. Assessing muscle strength using a dynamometer seems to be a promising method, as the reproducibility, validity, and sensitivity to change might be much better than MMT; on the other hand, it is largely undescribed for axial muscles. There are two options of dynamometric assessment, either using a fixed/isokinetic dynamometer or a handheld dynamometer (HHD). The isokinetic dynamometer is often considered to be the “gold standard” for measuring trunk muscle strength; however, it carries a high financial, space, and expert cost. A number of studies have been focused on the need to identify an alternative clinical tool that is practical, less expensive and more user-friendly, and that could provide reliable information on back extensor strength. The HHD has been shown to have all this, and its excellent validity and reliability were confirmed (27–30). In a previous study (14), we unified the methodology of the three existing protocols (27–30) using HHD to examine the maximal isometric lower back extensor strength in three different postures (prone, sitting, and standing). We verified that the repeatability and short- and long-term test–retest reliability of techniques for measuring maximal isometric strength of the lower back extensors using HHD are excellent in all three postures (14). In the current study, we used this technique to assess patients with MD2 and to compare them to healthy volunteers.

To create a complete picture of trunk muscle function in patients with MD2 we supplemented the battery of tests used in this study with tests examining not only the strength of back extensors but also the strength of respiratory muscles, which are likewise a part of the deep trunk muscle system, and also those examining the endurance of trunk muscles.

Using this battery of tests, we found significantly much lower isometric strength of the low back extensor muscles in patients with MD2 than in healthy volunteers. This supports the current knowledge that MD2 may be conducted as axial myopathy with prominent paraspinal involvement as a part of more widespread myopathy (9). In addition, using the novel battery of tests, we also revealed a significantly lower endurance in all tests examining trunk muscles (Biering-Sørensen, prone-plank, and side-bridge tests on both sides) in patients with MD2. To our knowledge, there is minimum information about trunk muscle endurance in axial myopathies. In our previous study of healthy individuals, we found that there was weak or no correlation between strength testing procedures and trunk muscular endurance tests (14). This finding suggests that even in healthy people there may be a difference between absolute strength and muscular endurance of the trunk muscles; both aspects should therefore be monitored. Another assessment tool from the test battery, respiratory muscle strength measurement, revealed significantly lower strength of both inspiratory and expiratory muscles in patients with MD2 than in HV.

The generally accepted pathophysiology of respiratory involvement in neuromuscular diseases is restriction of ventilation due to respiratory muscle weakness (31–34). Forced vital capacity (FVC) is an indicator of inspiratory muscle strength and is the most frequently used and well-studied test to evaluate respiration in neuromuscular disorders (NMD), as a decrease in FVC may reflect the presence of a restrictive ventilatory defect. However, some studies suggest that other pulmonary examinations may point out respiratory muscle weakness more promptly in patients with NMD. They concluded that FVC is not the best indicator of respiratory insufficiency; MIP ≤ 60 cm H2O is a more precise parameter (34–36). We assessed MIP and MEP in our patients, as this parameter has greater potential to catch discrete changes in particular respiratory muscle strength.

There is very limited documentation discussing the pathophysiology of respiratory involvement in MD2. This may be caused by the rareness of the subtype of the disease worldwide and because MD2 is considered to be a milder form of muscular dystrophy, without severe respiratory impairment. The respiratory muscle weakness in patients with MD2 that we observed in our study may have a variety of origins. As there is no evidence of a dystrophy process among the respiratory muscles in MD2 (37), the reason for the weakness seems to be either because of the overall decondition or a specific deconditioning of respiratory muscles. These are a component of the deep spinal (core) stabilization system, which is disabled in MD2 because of paraspinal muscle weakness. The next possible reason for low respiratory muscle performance could be myotonia of the respiratory muscles, which is however an unlikely phenomenon in patients with MD2. Myotonia of the respiratory muscles contributes to a chaotic breathing pattern and increases work in breathing (32). Respiratory muscle weakness and potentially myotonia of the respiratory muscles may produce discomfort in MD2 that has not yet been considered.

Non-specific (axial) low back pain (NLBP) is a widespread complaint; it is the most prevalent type of low back pain (LBP). It is reported to be a major health and socioeconomic problem that is accompanied by work absenteeism, disability, and high costs to patients and society. Although the underlying scientific evidence is limited, the best available estimates suggest that the prevalence of chronic NLBP (CNLBP) in the general population is approximately 23% (38–40).

In the current study, more than half of the patients with MD2 (21 out of 40, 52.5%) had CLBP and axial pain predominantly. This is somewhat less than in another study of the prevalence of back pain in patients with axial myopathy in which the number came up to 66% (13). The slightly lower number of patients with CLBP in our study may be due to the selection method of MD2 patients: one exclusion criterion was muscle strength of hip extensors less than 4. This criterion was based on one of the functional assessments, Biering-Sørensen test, which challenges not only lower back extensor muscles but also hip extensors. In case of hip extensor weakness, a different Biering-Sørensen test protocol would need to be employed. For the sake of measurement consistency, we set the exclusion criterion declared above. This may have brought us somewhat less severely afflicted patients. MD2 patients with more pronounced muscle weakness can also be expected to have more frequent CLBP.

The MD2 patients in the current study with CLBP exhibited lower maximal isometric strength of lower back extensors as measured by HHD than the subgroup without CLBP. Interestingly, the difference in lower back extensor strength was not significant between the two subgroups of MD2 patients using manual muscle testing rating by means of the MRC scale. This indicates the need for quantitative muscle testing, to be able to differentiate minor deviations that may be significant in clinical status. Significant differences in performance between those two MD2 subgroups were also found in the Biering-Sørensen test assessing the endurance of trunk muscle extensors and the side-bridge test on the right side. The same test performed on the left side proved borderline significant differences between the two subgroups, however, this may be due to the small number of participants in each group. A borderline significant difference was also found in physical activity expressed in MET, indicating that less physically active patients were more likely to have CLBP.

ROC analysis of potential risk factors for CLBP in patients with MD2 disclosed a number of parameters as effective discriminating factors between the two subgroups. Finally, multivariate logistic regression showed weakness of lower back extensors (specifically maximal isometric lower back extensor strength in prone position ≤ 15.8 kg) as an independent risk factor for the occurrence of CLBP in MD2 patients; other possible risk factors were myotonia severity (Myotonia Behavior Scale ≥ 2) and lower physical activity (MET ≤ 1,272 min per week) but the statistical significance of these two factors was borderline, which may have been due to the smaller number of patients.

The literature concerning neuromuscular disorders acknowledges that decreased axial musculature strength may be a cause of pain; however, this potential factor is still under discussion (9, 30). It has been reported that inadequate function of the lumbar extensor muscles and poor coordination of core muscles are considered important etiological factors in low back pain in the general population (10, 41–44). Specific lumbar extensor deconditioning is by no means considered the only causal factor in CLBP, which is recognized as a multifactorial condition (44). Additional risk factors for the development of back pain and its chronification vary between studies. A number of environmental and individual characteristics have been found to increase the risk of LBP. An umbrella review of evidence from available systematic reviews was carried out to provide an overview of risk factors for LBP (45). A total of 38 risk factors were significantly associated with enhanced risk of LBP or sciatica in at least one systematic review; the odds ratios ranged from 1.26 to 13.00. Adverse risk factors included individual characteristics (e.g., older age, previous low back pain, greater height), poor general health (e.g., smoking, obesity, chronic diseases, sleep problems, pain at any other regional site), physical stress on spine [e.g., whole-body vibration, prolonged standing or walking (> 2 h), time driving (>2 h), bending forward and backward (often)], and psychological stress (e.g., monotonous work, mental distress, dissatisfaction with life, depression, psychosomatic factors) (45). According to a study by Nieminen et al., several prognostic factors, especially from the biomechanical, psychological, and psychosocial viewpoints, are significant for chronicity in low back pain (46). Many of these risk factors for CLBP have been analyzed in our patients with MD2 (e.g., age, smoking, BMI, depression, presence of chronic musculoskeletal pain except LBP), but their influence was not confirmed in this cohort, probably due to the fact that this is not the general population but patients with a muscular disease where trunk muscle dysfunction comes to the fore. It is possible that central pain mechanisms such as central sensitization and dysfunction of the descending pain inhibitory system are also involved in the development of CLBP in MD2 patients. It is very likely that these mechanisms play a role in the development of muscle pain in these patients. On the other hand, in our cohort, the presence of chronic musculoskeletal pain (except low back pain) was not a risk factor for CLBP; therefore, we conclude that in patients with CLBP, trunk muscle dysfunction is more involved in the development of low back pain than central pain mechanisms.

The present study has some limitations. The most significant limitation is the relatively low number of patients with MD2, which may affect the statistical significance of the results and impact especially on determining risk factors and OR values. However, MD2 is an orphan disease and large patient cohorts cannot be expected. Another limitation is that the result of CLBP prevalence in MD2 patients in this study cannot be fully generalized to the entire MD2 population because this was a selected cohort and the study exclusion criteria favored less functionally impaired patients. It is also important to take into account, that in this study females predominated in the MD2 patient group due to their greater compliance and willingness to participate in the study, and at the same time women are more likely to experience chronic pain, including CLBP, than men. The question remains whether the trunk muscle weakness verified by the current study is the reason for CLBP in these patients or the consequence of the pain that is a relevant problem in this patient population. The pain may cause decreased overall activity, leading to further weakening; the weakness may be the source of pain, creating a vicious circle. On the other hand, this question applies to back pain in general, not only to MD2 patients, and it is being studied in the general population. It can be assumed that an important approach in the treatment of CLBP is to interrupt this vicious circle, for example by improving the function of the deep spinal stabilization system with appropriate rehabilitation and physical activity. Conversely, a positive approach of this study is that we compared trunk muscle strength and endurance in MD2 patients with healthy controls who were selected to match age, gender, and BMI as closely as possible, as functional parameters of trunk muscles may be influenced by these physiological variables. A contribution of this study is the comprehensive clinical examination of both MD2 patients and healthy volunteers with a very detailed assessment of trunk muscle function and the evaluation of the presence of CLBP in patients with MD2 together with risk factors, which to our knowledge has not been evaluated before.

We believe that the findings of this study may be beneficial for managing patients with MD2 in routine clinical practice. It seems appropriate for these patients to strengthen the respiratory muscles (e.g., inspiratory muscle training using a Threshold IMT device and positive expiratory pressure training using a Threshold PEP device). We assume that strengthening the lower back extensor muscles, improving deep stabilization system coordination, and performing regular aerobic activity (e.g., regular walks and swimming) could help reduce the occurrence of chronic low back pain in patients with MD2.

## 5. Conclusion

Patients with MD2 showed significant dysfunction in their trunk muscles, including the respiratory muscles, expressed by decreased muscle strength and endurance. In a subgroup of MD2 patients with CLBP, the lower back extensor dysfunction was pronounced. Decreased muscle strength of the lower back extensors together with a possible contribution of myotonia severity and reduced physical activity appear to be risk factors for the frequent occurrence of CLBP in patients with MD2. These findings should be considered in management and rehabilitation planning in patients with MD2.

## Data availability statement

The raw data supporting the conclusions of this article will be made available by the authors, without undue reservation.

## Ethics statement

The studies involving humans were approved by Ethics Committee—University Hospital Brno (agreement number 05-090621/EK). The studies were conducted in accordance with the local legislation and institutional requirements. The participants provided their written informed consent to participate in this study.

## Author contributions

DV: Conceptualization, Methodology, Project administration, Writing – original draft, Writing – review & editing, Data curation, Investigation. PK: Conceptualization, Data curation, Investigation, Writing – original draft, Writing – review & editing, Project administration. MS: Investigation, Methodology, Writing – review & editing. OP: Investigation, Writing – review & editing. TB: Data curation, Visualization, Writing – review & editing. KH: Data curation, Visualization, Writing – review & editing. SV: Funding acquisition, Resources, Writing – review & editing. KM: Investigation, Writing – review & editing. BA: Conceptualization, Data curation, Formal analysis, Funding acquisition, Investigation, Methodology, Project administration, Resources, Supervision, Validation, Writing – original draft, Writing – review & editing.
